# Supplementary material for: Temperature-Driven Transformation of CsPbBr3 Nanoplatelets into Mosaic Nanotiles in Solution through Self-Assembly
Source: Nano Lett. 2020 Jan 28;20(3):1808–18. doi: 10.1021/acs.nanolett.9b05036 (PMC7997623; doi:10.1021/acs.nanolett.9b05036)
Supplement: Supplementary file 1 — nl9b05036_si_001.pdf [file nl9b05036_si_001.pdf]

Supporting information for

Temperature Driven Transformation of $CsPbBr_3$ Nanoplatelets into Mosaic Nanotiles in Solution through Self-Assembly

Zhiya Dang[†], Balaji Dhanabalan[†ɫ], Andrea Castelli[†ძ], Rohan Dhall[¥], Karen C. Bustillo[¥], Dorwal Marchelli[‡], Davide Spirito[‡ძ], Urko Petralanda[†], Javad Shamsi[†§], Liberato Manna[†*], Roman Krahne[‡], and Milena P. Arciniegas[†*]

[†]Nanochemistry Department. [‡]Optoelectronics. Istituto Italiano di Tecnologia, Via Morego 30, 16163 Genova, Italy.

[ɫ]Dipartimento di Chimica e Chimica Industriale, Università degli Studi di Genova, Via Dodecaneso, 31, 16146 Genova, Italy.

[¥]National Center for Electron Microscopy, Molecular Foundry, Lawrence Berkeley National Laboratory, Berkeley, California 94720, United States.

1. **TEM details of the starting NPL stacks and objects at different stages of the transformation.**

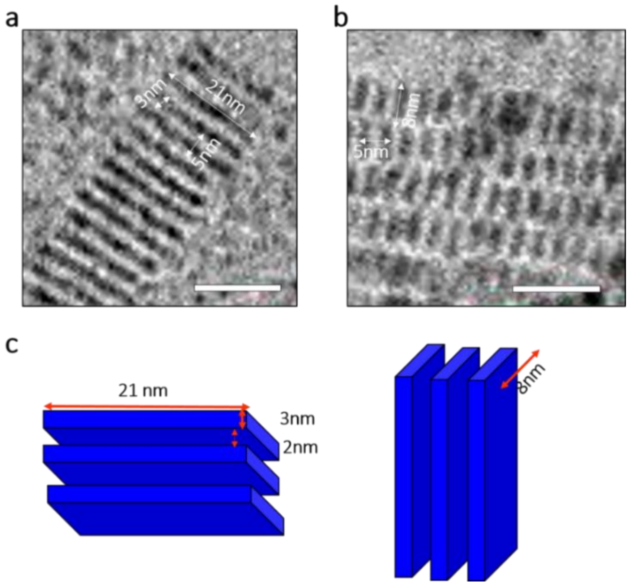

Figure S1. (a-b) Magnified TEM images of the as-synthesized $CsPbBr_3$ NPL stacks showing the two possible orientations of the individual NPL in the stacks with respect to the substrate: standing on their long (a) or short lateral (b) side, as highlighted in the corresponding sketches in (c). Scale bars: 20 nm.

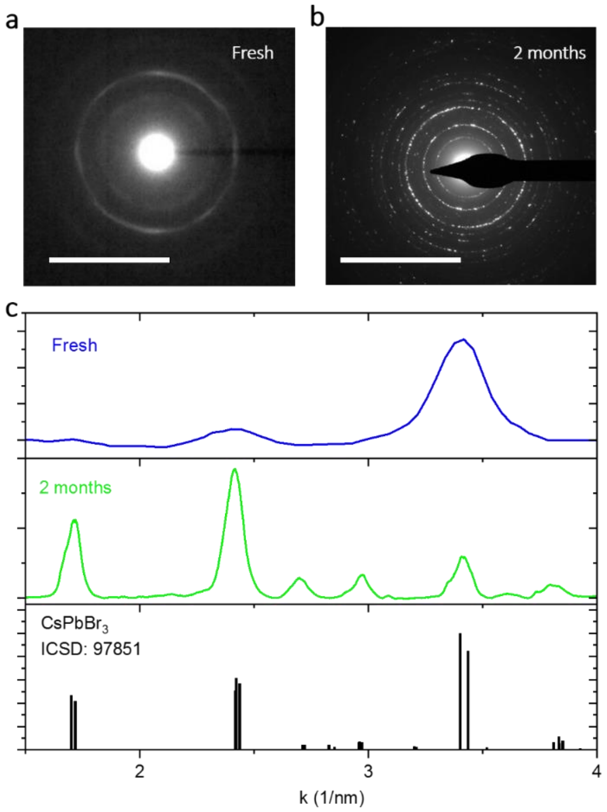

Figure S2. (a-b) SAED pattern of the fresh NPLs and of the nanotiles formed after two-months (Scale bar: 5 1/nm); (c) Azimuthal integration of the SAED patterns shown in (a) and (b), and their comparison with reference to the orthorhombic $CsPbBr_3$ phase (ICSD 97851 pattern in black). The results show that the structure of both nanocrystals correspond to an orthorhombic $CsPbBr_3$ phase, with broader peaks from the fresh NPLs due to their smaller size compared to the nanotiles.

Table S1. Average size (in terms of projected area in TEM images) and number of structures observed at different times of the transformation in the field of view (FOV), as measured via TEM analysis of overview images.

**Aging time**	**Average number of structures per μm^2**	**Average size, nm^2**
0	3480 ± 332 Nanoplatelets	63 ± 4
1 week	878 ± 63	546
1 month	209 ± 15 belts, 1871 ± 348 NPLs	1624 for belts; 63 for NPLs
2 months	8 ± 3 nanotiles	34500

After one week, structures with different size are present in the solutions, as observed in Figure 1b of the main text and Figure S5. After one month, there is a growing population of objects made of nanobelts and a reduced number of the initial NPLs. The analysis in Table S1 was performed on four different regions from three different TEM grids. Representative overview TEM images used for the analysis are shown in Figures S4-S7.

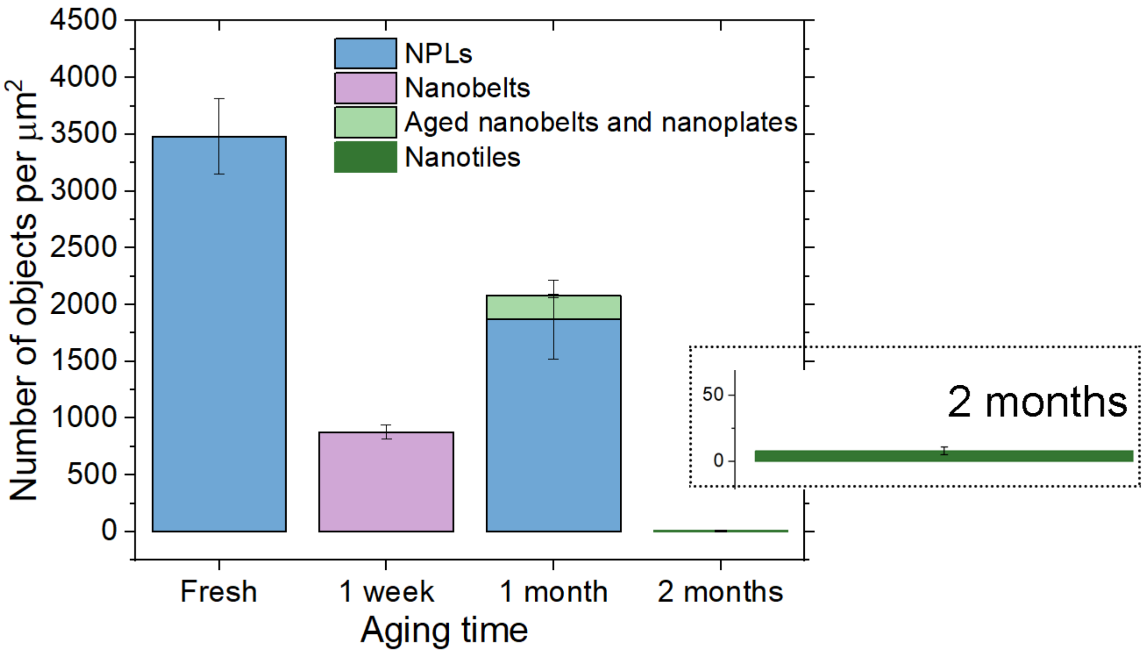

Figure S3. Average number of objects per μm^2 as a function of the aging time up to 2 months. Clearly, there is a reduction of the number of NPLs that is accompanied by an increased number of nanobelts. After two months (zoomed portion of the plot framed with dotted lines) only few nanotiles with large size (see Table S1) are observed.

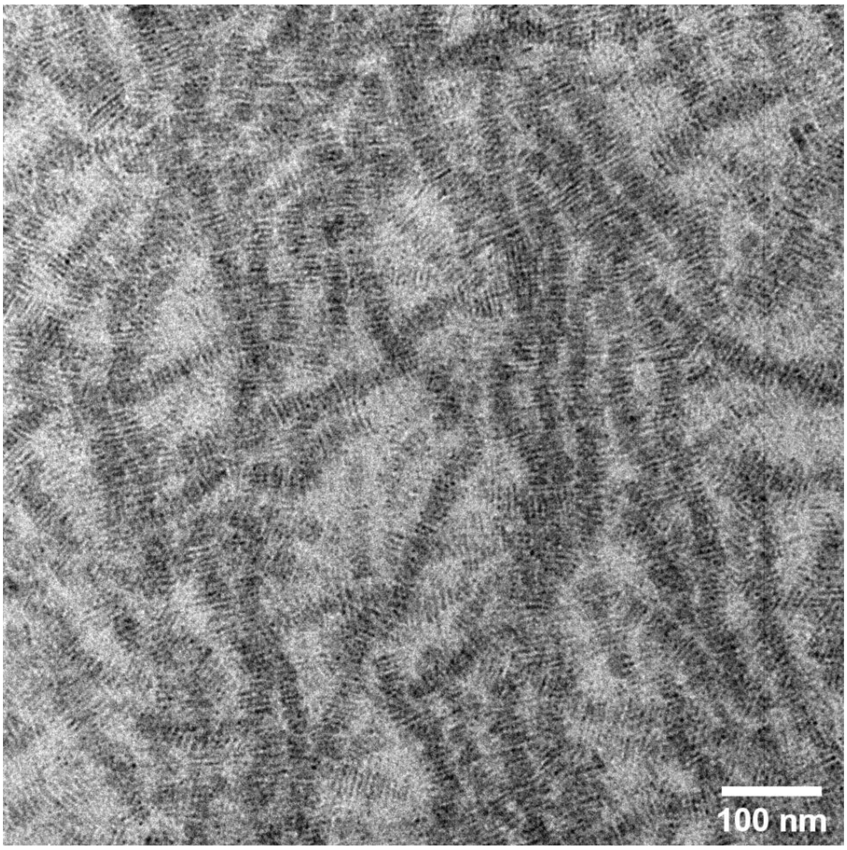

Figure S4. Representative overview TEM image of the as-synthesized $CsPbBr_3$ NPLs in an area of 0.8 x 0.8 μm^2

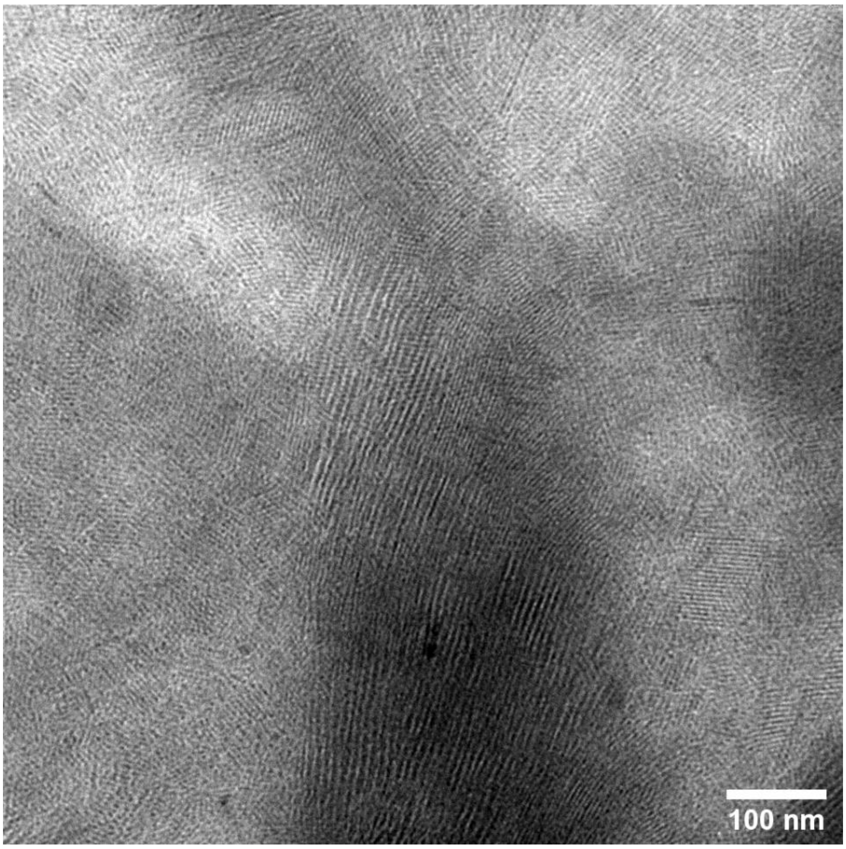

Figure S5. Representative overview TEM image of $CsPbBr_3$ NPLs after one week of aging.

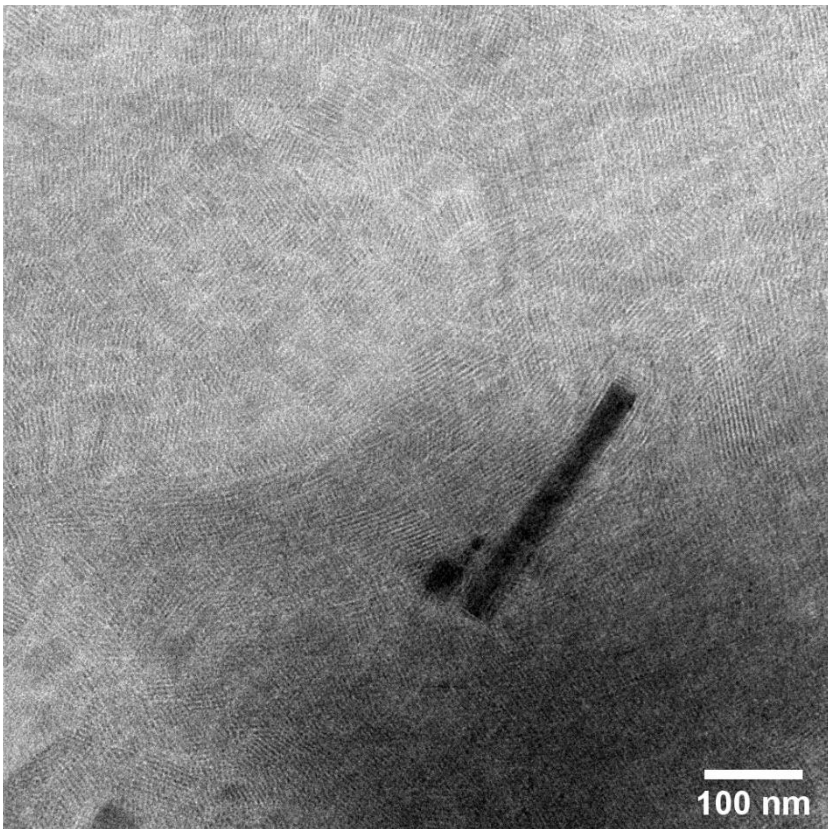

Figure S6. Representative overview TEM image taken after 1 month of aging. A short and a long nanobelt, surrounded by smaller ones, and a few NPLs, are observed.

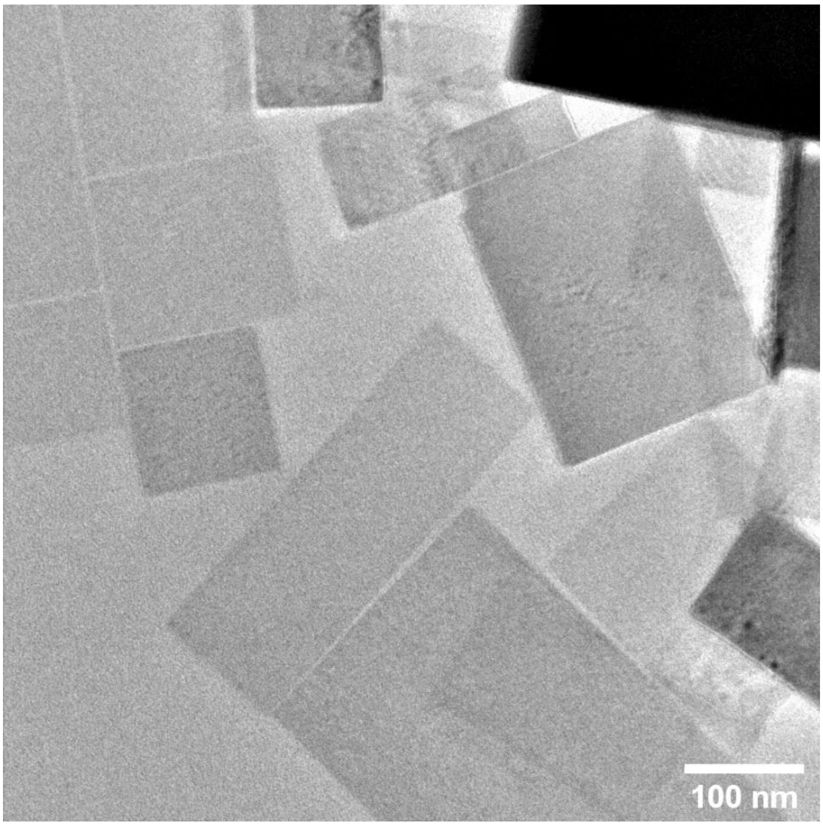

Figure S7. Representative overview TEM image recorded after 2 months of aging of the NPL solution. Only nanotiles are observed in all the inspected regions from different samples.

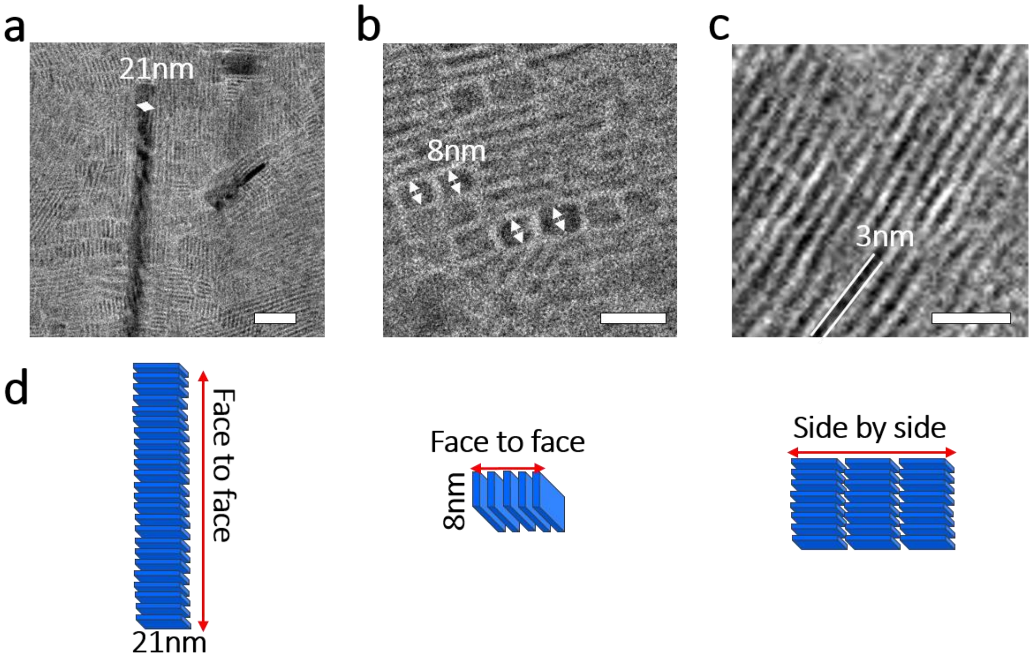

Figure S8. TEM images of nanobelts formed after short time of aging: (a) long-range face-to-face merging of NPL stacks forming a nanobelt. Scale bar: 50 nm; (b) short-range face-to-face merging of NPL stacks that develop into a short nanobelt; (c) nanobelt assemblies formed by side-to-side merging of NPL stacks. Scale bars (b-c): 20 nm. (d) A set of sketches showing from left to right the corresponding configurations observed in the TEM images.

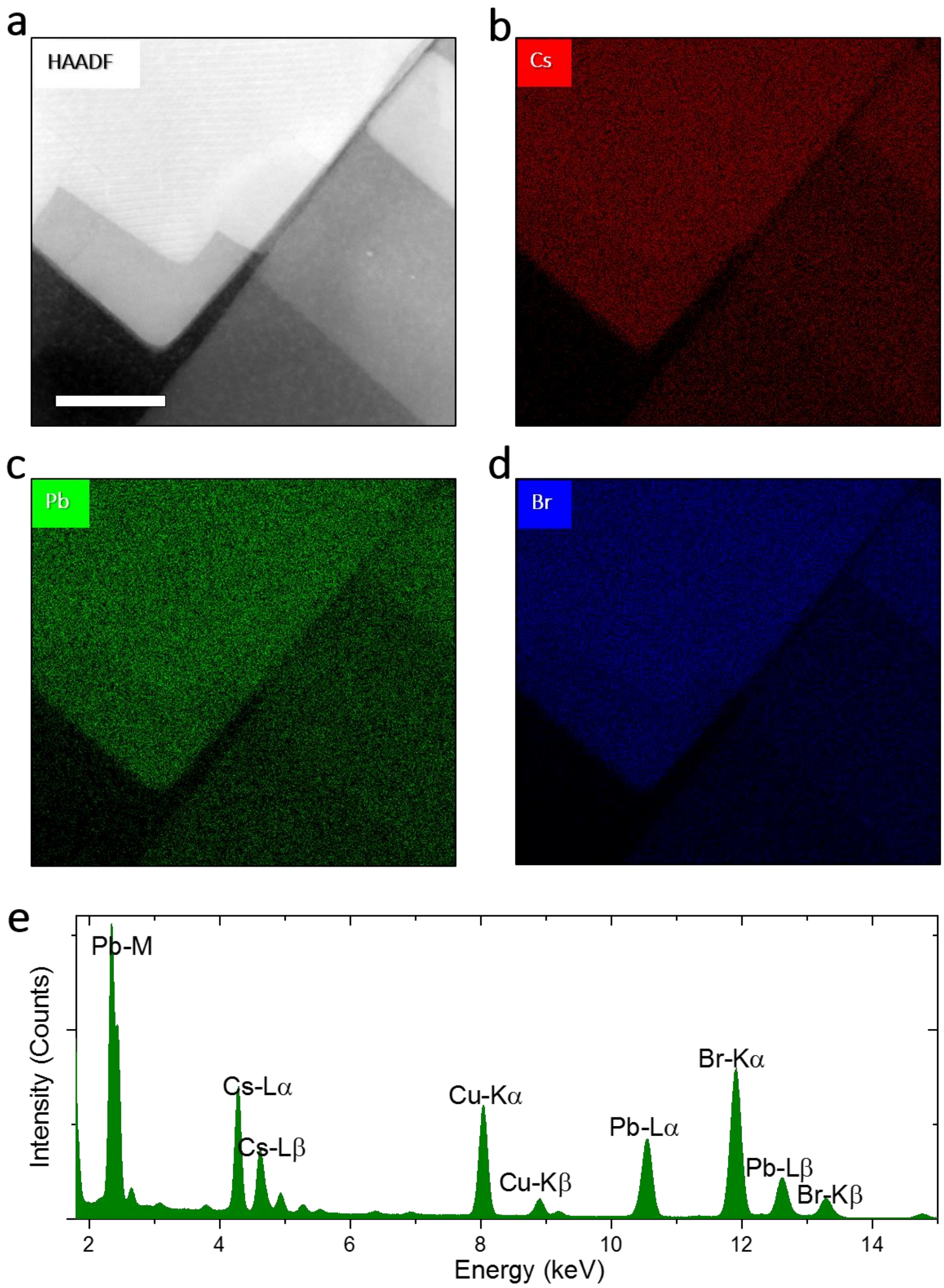

Figure S9. (a) HAADF-STEM image of final nanotiles and their corresponding EDS elemental maps for Cs (b), Pb (c), and Br (d). Scale bar: 50 nm. (e) Collected EDS spectrum from the region shown in (a).

Table S2. Compositional analysis of the final nanotiles displayed in Figure S9.

Element	Atomic Fraction (%)	Atomic Error (%)
Br	60.83	5.61
Cs	19.5	2.52
Pb	19.67	2.55

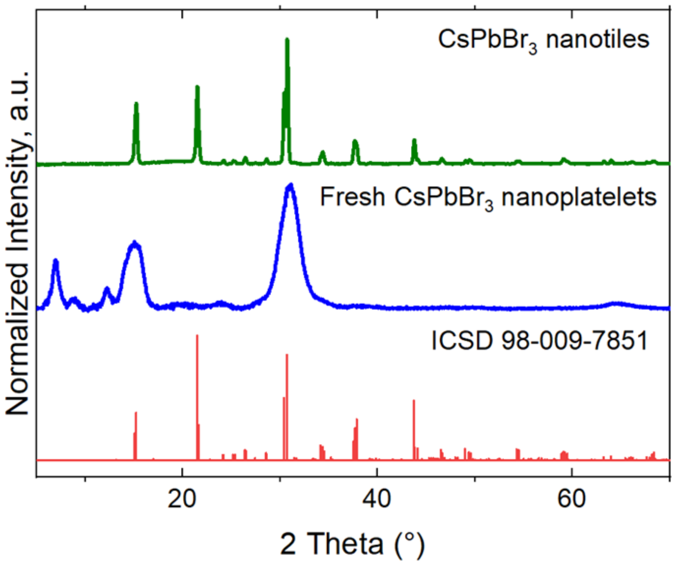

Figure S10. XRD patterns collected from a diluted sample of the freshly synthesized $CsPbBr_3$ nanoplatelets and compared to 2-months aged samples, the so called nanotiles. The XRD reference pattern for the $CsPbBr_3$ orthorhombic crystallographic structures is included.

Table S3 reports the average lateral crystallite domain size of the $CsPbBr_3$ nanotiles calculated by using the Scherrer equation (with a K = 0.94), after performing the line profile analysis of the XRD patterns to determine the representative full width at half maximum (FWHM) of the selected diffraction peaks. Taking the orthorhombic crystal structure of the nanotiles into account, we have used the (002) planes for estimating the average lateral size of the domains. In the case of the NPLs, we have performed a similar analysis by using the SAED pattern shown in Figure S2b. We have calculated a value of the nanoplate lateral size of ~ 22 nm, which is close to the estimated value of 21 nm from TEM image analysis. Note that it was not technically possible to perform the line profile analysis on the XRD pattern collected from as-synthesized (highly concentrated) NPLs due to the presence of highly intense diffraction peaks related to their oriented assemblies, which were close to those needed for the analysis. The XRD pattern in Figure S10 was collected from a highly diluted suspension of NPLs. In this case, however, they preferentially lay down on their basal facet, parallel to the substrate, and thus, the observed diffraction peaks are associated to their thickness, of ca. 3.7 nm. The reported values were obtained after instrumental broadening correction.

Table S3. Crystalline domain size obtained via XRD line narrowing analysis. L: lateral size and t: thickness.

	**hkl**	**Peak position, 2ϴ**	**FWHM, rad**	**FWHM LaB6 (Instrumental broadening)**	**Scherrer calculation, nm**	**Crystallite size after broadening correction, nm**
**Nanotiles**						
L and t	110	15.2239	0.2376	0.063	33.7	46
L	020	21.5265	0.1574	0.062	51.4	85
L	200	21.6630	0.1828	0.062	44.2	67
L	**002**	15.0617	0.1751	0.062	45.8	70
**NPLs**						
t	020	30.9678	2.2632	0.0517	3.6	3.7

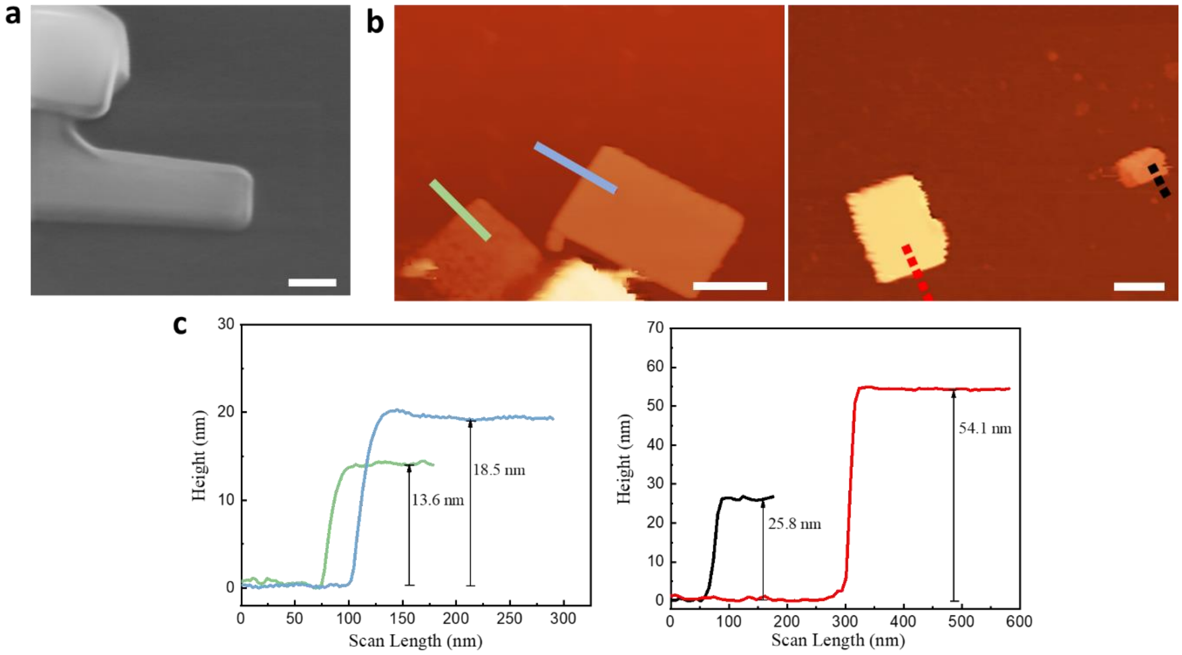

Figure S11. Height analysis of the nanotiles deposited by spin coating on Si substrates**.** (a) SEM image of two stacked nanotiles with ca. 40 nm thickness recorded at a tilting angle of 50°. Scale bar: 100 nm. (b-c) Contact mode AFM images of nanotiles (in b), together with the height profiles (c) acquired along the colored lines in (b). Scale bars: 250 nm.

2. Optical properties of the structures at different stages of the ageing.

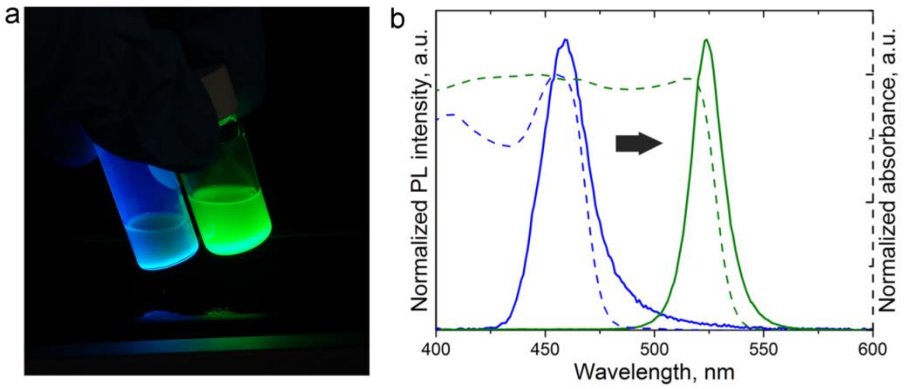

Figure S12. (a) Photograph taken under UV lamp illumination of blue-emitting fresh NPLs and a 2-months aged solution of NPLs. (b) PL (solid lines) and absorption (dashed lines) spectra acquired from the as-synthesized $CsPbBr_3$ NPL stacks in solution (blue) and from a 2-months aged solution (green) that show an emission peaks centered at 460 nm with a full width at half maximum (FWHM) of 27 nm and 525 nm with a FWHM of 18 nm, respectively.

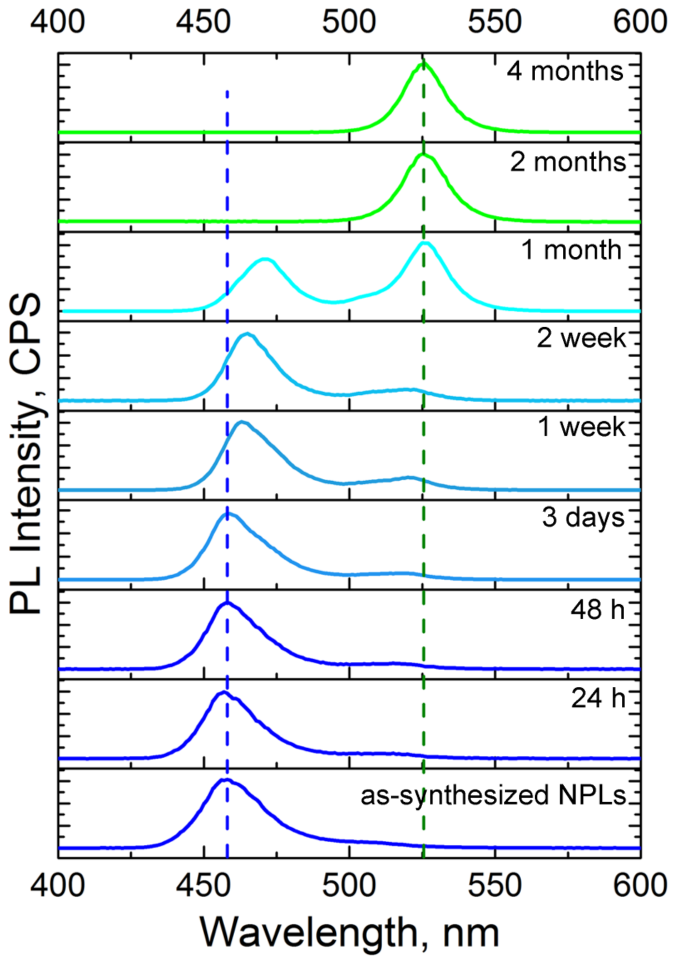

Figure S13. PL recorded at different times from a fresh (labeled as "as-synthesized NPLs") to a four months-aged solution showing the evolution of the emission. After one week a red-shift of the emission peak at 458 nm (indicated with a blue-dashed line) to 462 nm occurs, acompanied by the formation of a second emission peak with a maximum at 520 nm that intensified over time and shifted to 525 nm (indicated with a green-dashed line).

3. Compositional analysis of hexagonal-shaped nanocrystals in aged solutions.

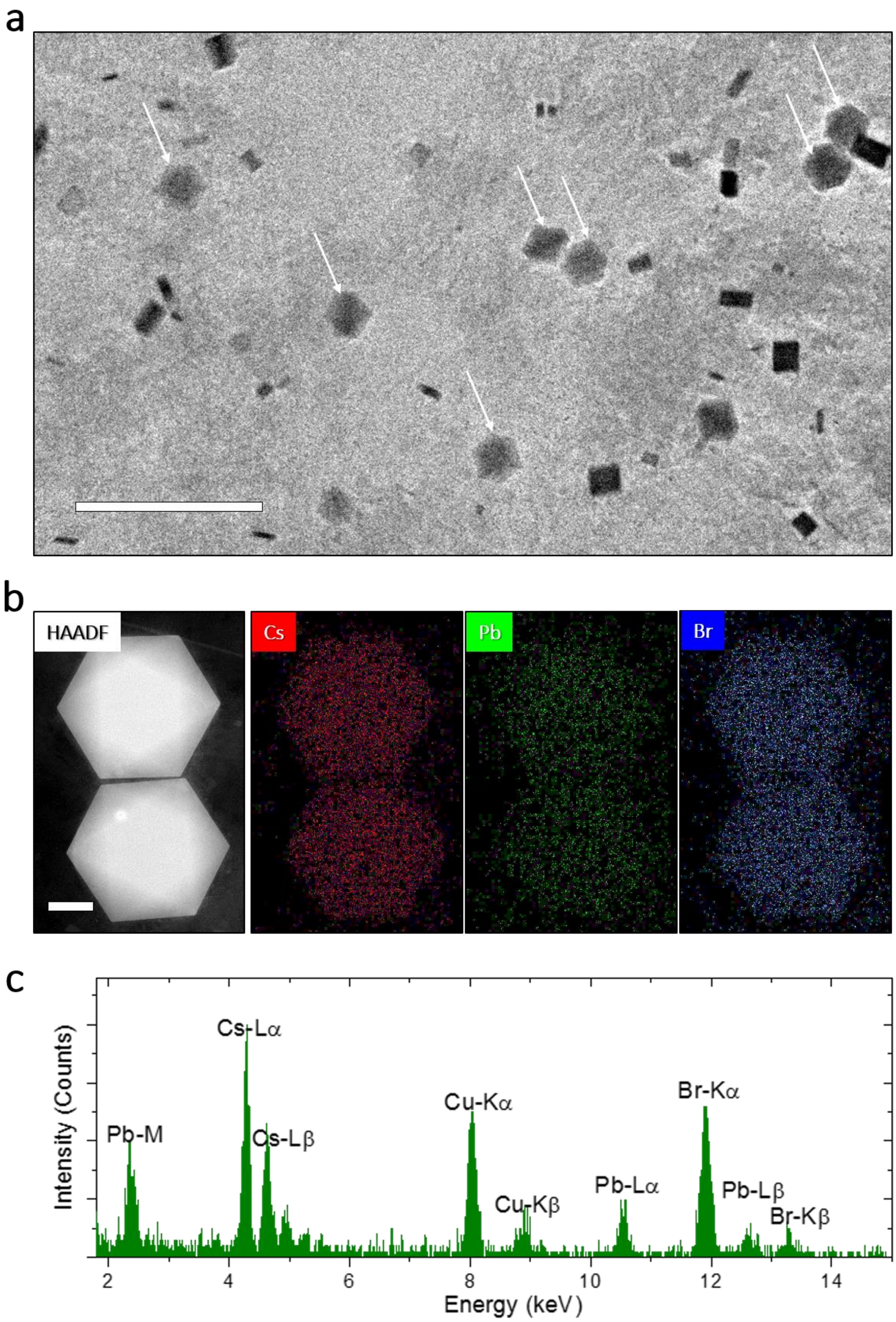

Figure S14. (a) TEM overview image of hexagonal-shaped nanocrystals (indicated by white arrows) that were found randomly dispersed among the initial stacks in 1 week-aged solution. (b) HAADF-STEM image of two of the hexagonal structures and their corresponding EDS elemental maps for Cs (in red), Pb (in green), and Br (in blue). (c) Collected EDS spectrum from the region shown in (b). Scale bars: 2 µm (a); 200 nm (b).

Table S4. Composition analysis of the hexagonal shaped nanocrystals displayed in Figure S12.

Element	Atomic Fraction (%)	Atomic Error (%)
Br	61.04	5.79
Cs	30.21	3.96
Pb	8.75	1.17

4. Supplementary details on the FTIR analysis.

Table S5. List of assigned main vibrational peaks[1-3] observed in the FTIR spectra of the fresh and aged NPL solutions that are shown in Figure 2c of the main document.

Peak position (cm^{-1})	Assigned vibrational mode	Assigned ligand marker
3500	N-H wagging	Amine
3075	=C-H_2 stretching	ODE
3005	=C-H stretching	Oleates, amines
2940	C-H_3 asymmetric stretching	All
2925	C-H_2 asymmetric stretching	All
2855	C-H_2 symmetric stretching	All
1710	C=O stretching	OA
1645	C=C stretching	ODE
1605-1585	N-H bending	Amines, ammoniums
1540	COO^- asymmetric stretching	Cs-oleate
1490	N-H bending	Ammoniums
1465	C-H_2 bending (scissor)	All
1405	COO- symmetric stretching	Pb-oleate
1040	C-N stretching	Amines, ammoniums
930	COO-H bending	OA
900	=C-H wagging	ODE
800	N-H wagging	Amine
725	C-H_2 bending	All

5. **Additional examples of aligned and imperfect attachment between adjacent domains.**

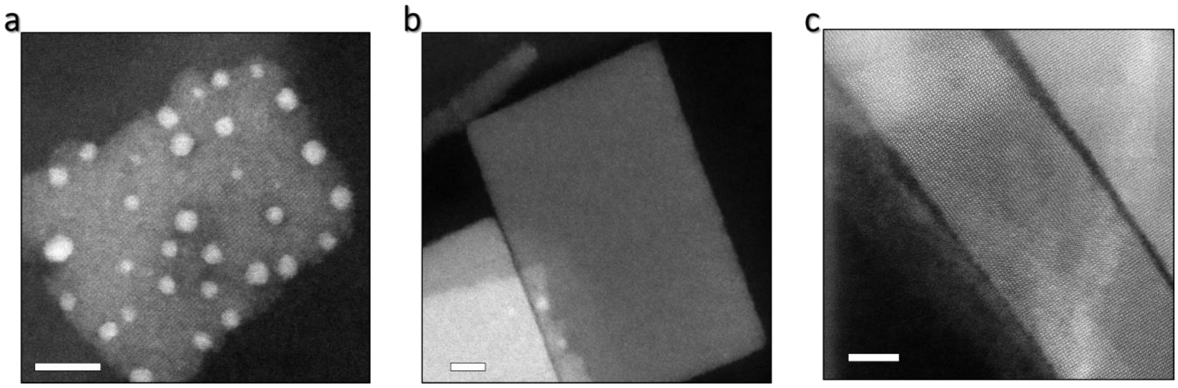

Figure S15. (a-c) HAADF-STEM images of nanobelts and nanoplates observed in different regions of a 2 weeks-aged solution showing that all nanobelts and nanoplates at this stage have continuous atomic lattices. The white dots observed in (a) are the result of an electron-beam induced reduction of Pb^{2+} and aggregation of Pb atoms, an event that occurs while imaging perovskite $CsPbBr_3$ nanocrystals, as demonstrated in a previous work from our group.[4]

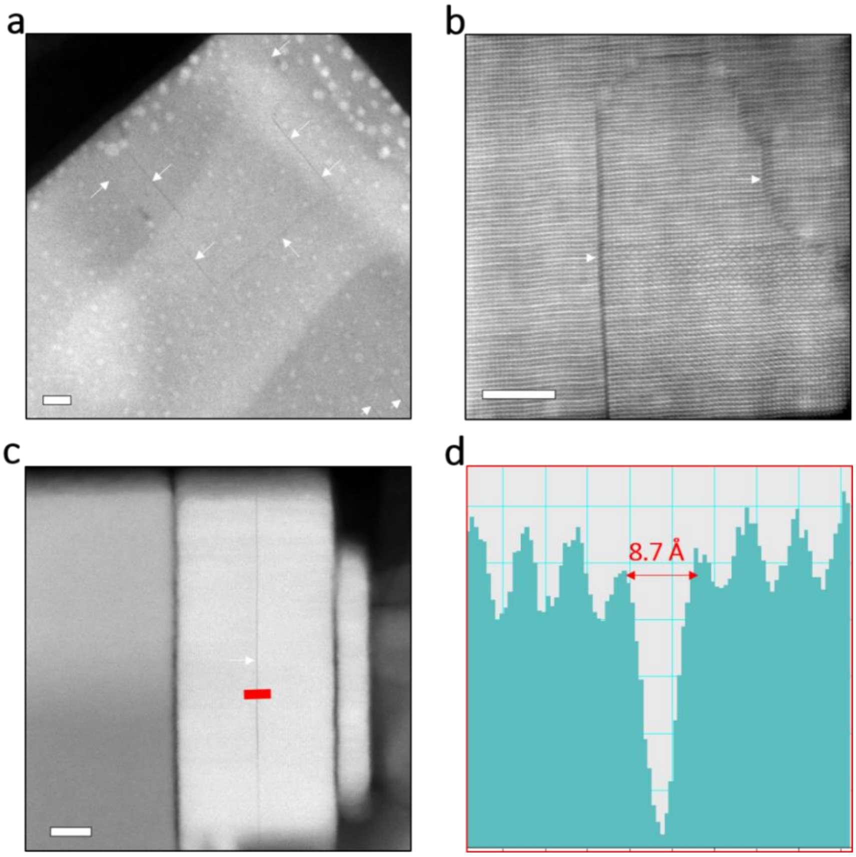

Figure S16. (a-c) HAADF-STEM images collected from different regions of a 2 months-aged solution containing large nanotiles made by the attachment of different components over time, which match their atoms at the boundaries (indicated with white arrows) imperfectly. (d) The line profile of intensity across the boundary indicated by red line in (c) shows a space between adjacent Pb-Br atomic columns of ca. 8.7 Å. Scale bars: 10 nm.

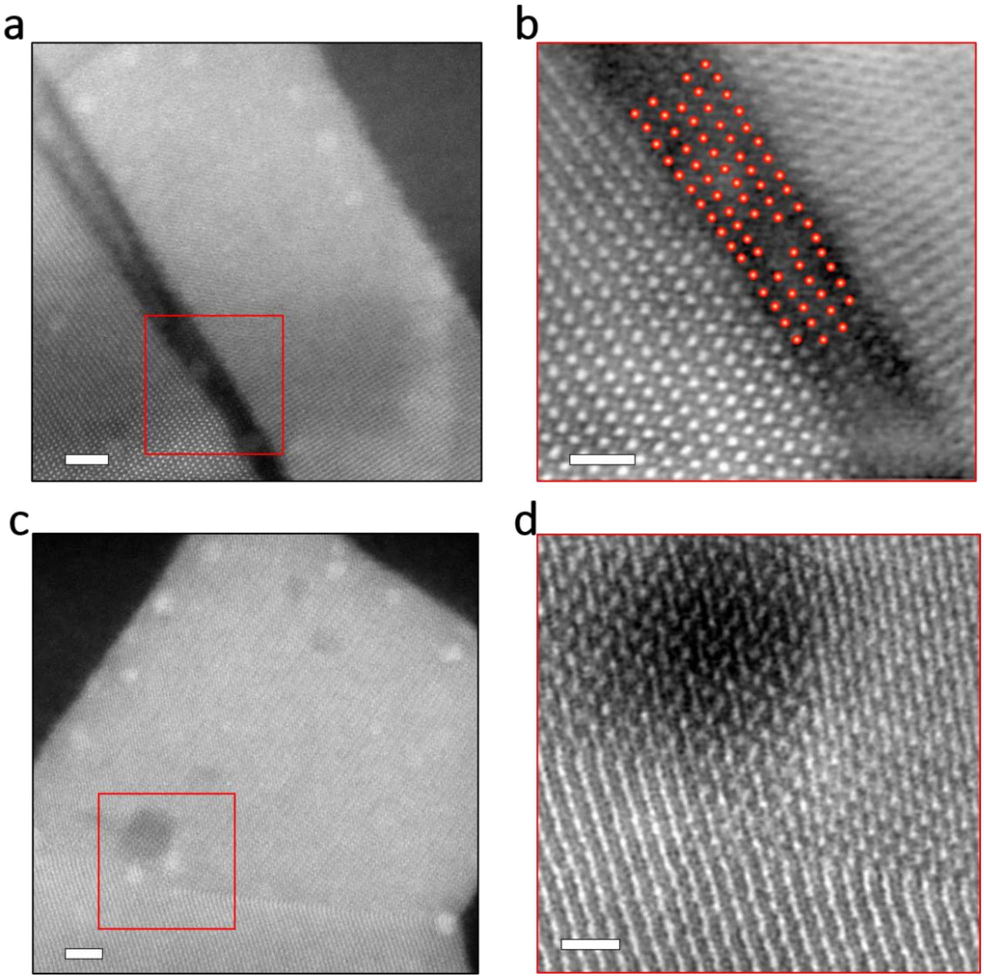

Figure S17. Collection of HAADF-STEM images showing other types of defects observed from aged NPL solutions: (a-b) dislocations and (c-d) grain boundaries. Scale bars (a, c): 5 nm; (b, d): 2 nm.

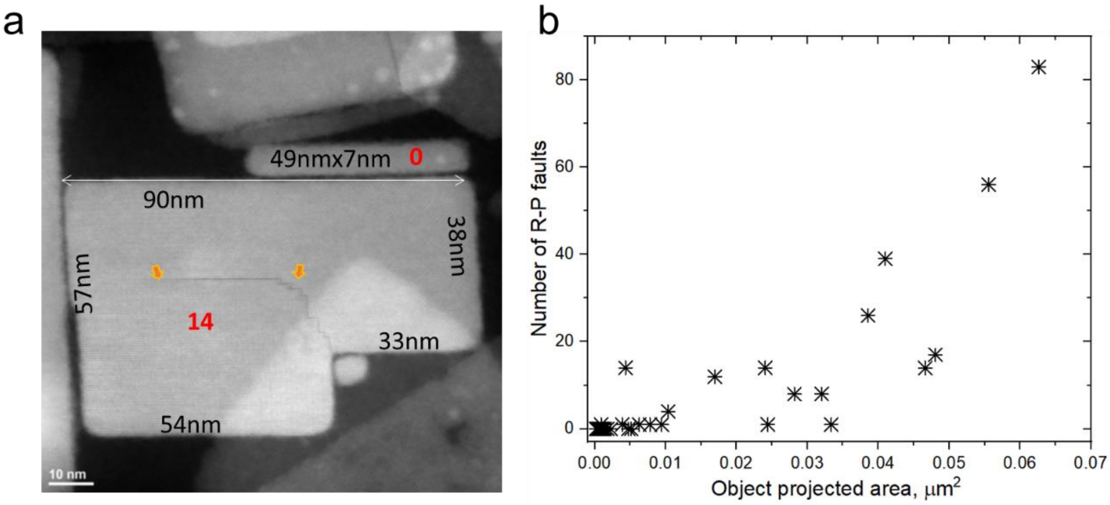

Figure S18. (a) HAADF-STEM image of a nanobelt without R-P faults close to a nanotile with around 14 R-P faults. (b) Number of R-P faults observed in the TEM projected areas. R-P faults are observed only on large objects. The counting of R-P faults was performed on structures observed from aliquots taken from solutions at different time of aging (1 and two months).

Details of the DFT computational analysis.

Table S6. Summary of the DFT calculation results obtained for the energy difference between two merged/attached structures with and without R-P planar faults (atomic column shift) and of different sizes, from 2x2 to 4x4 unit cells. The resulting energy difference as a function of the size of the merged domains shows that large structures require less energy for the formation of an imperfect atomic attachment than for an aligned one.

No shift	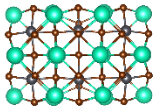	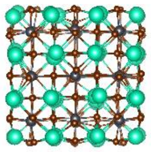	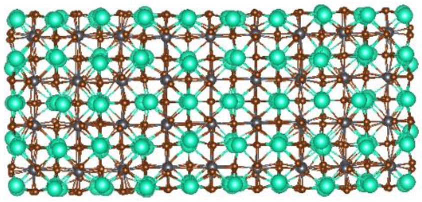
Shift ½ unit cell	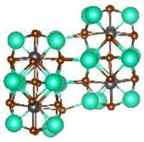	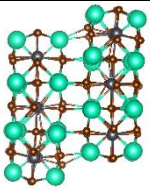	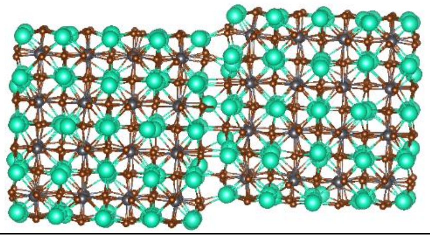
No. of unit cells	2x2	3x3	4x4
$\Delta \boldsymbol{E}$(eV/ Å ^{2})	0.29	0.235	0.21

6. Temperature-induced changes on the morphology of the as-synthesized NPLs stacks

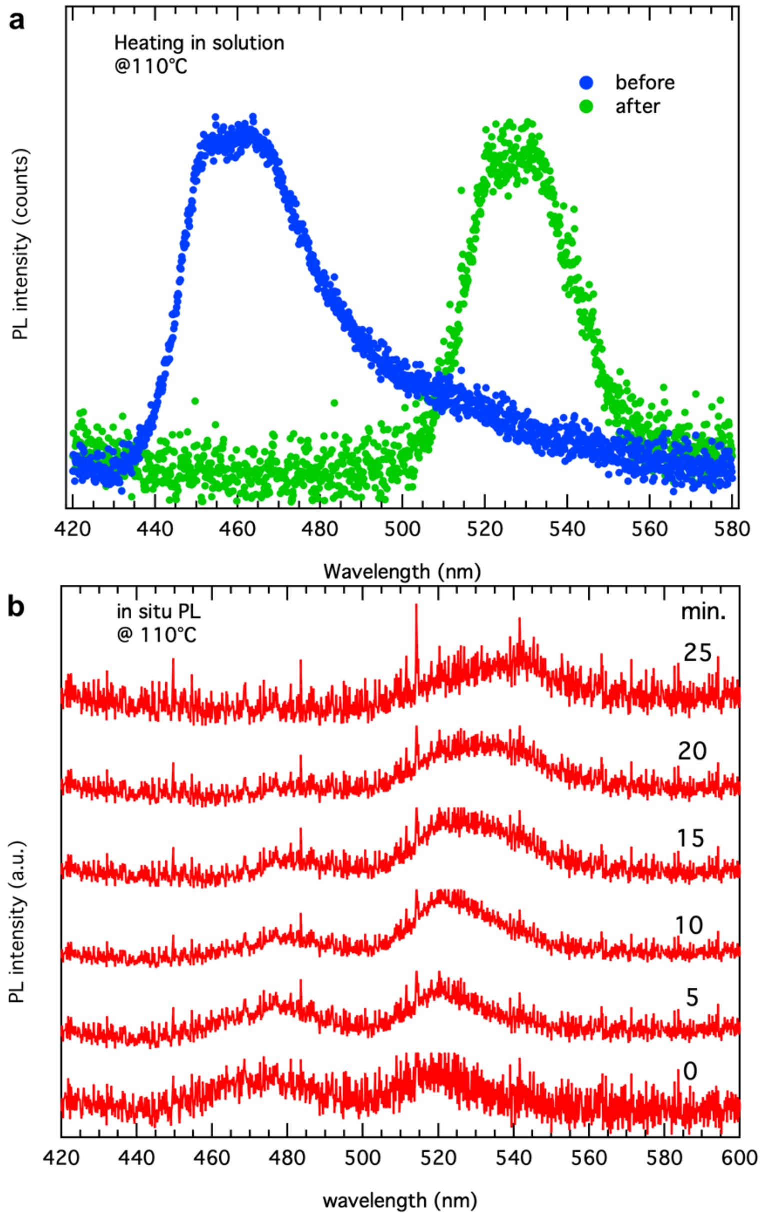

Figure S19. (a) PL recorded from a NPL solution before heating and after heating it to 110°C for 30 min. (b) PL spectra recorded in situ over time from a NPL suspension in toluene that was kept at a temperature of 110 °C. The signal-to-noise ratio is relatively low because the PL intensity is reduced at 110 °C. Furthermore, the solution was stirred to maintain all nanocrystal populations in the section of the vial that was optically probed.

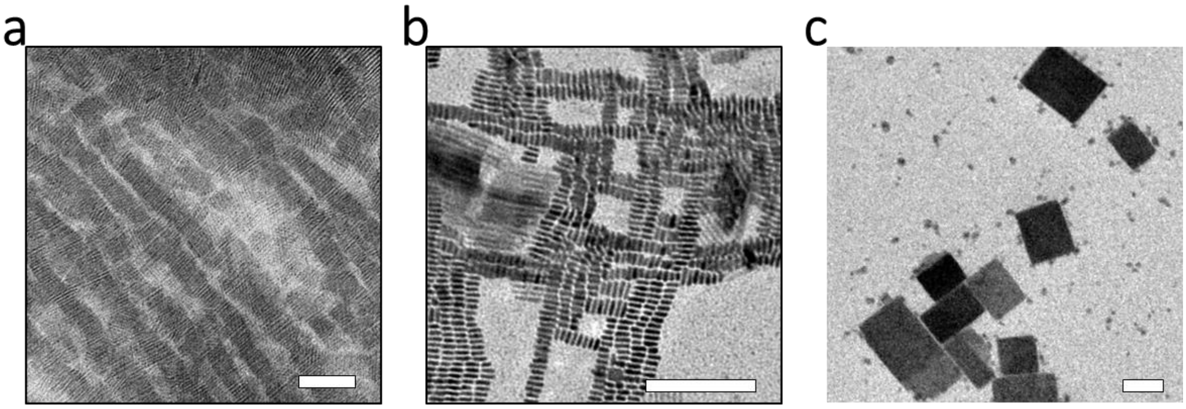

Figure S20. TEM images of (a) as-synthesized $CsPbBr_3$ NPL stacks and after heating them up at 50 °C for 1 hour (b) and 110 °C for 30 min (c). Scale bars: 100 nm.

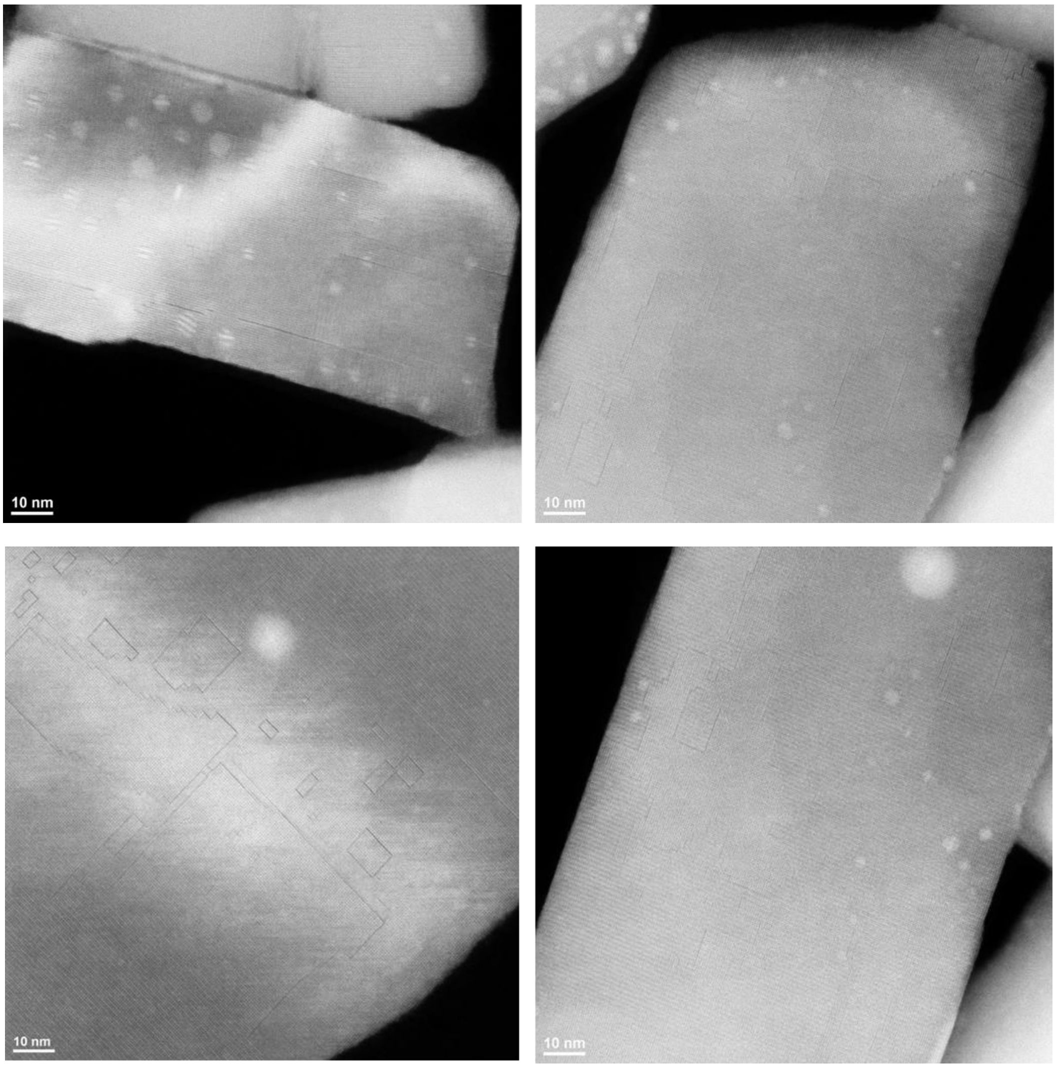

Figure S21. HAADF-STEM images showing the abundance of R-P faults observed in the nanotiles produced by heating the NPL solution at 110° C for 30 min. Scale bars: 10 nm.

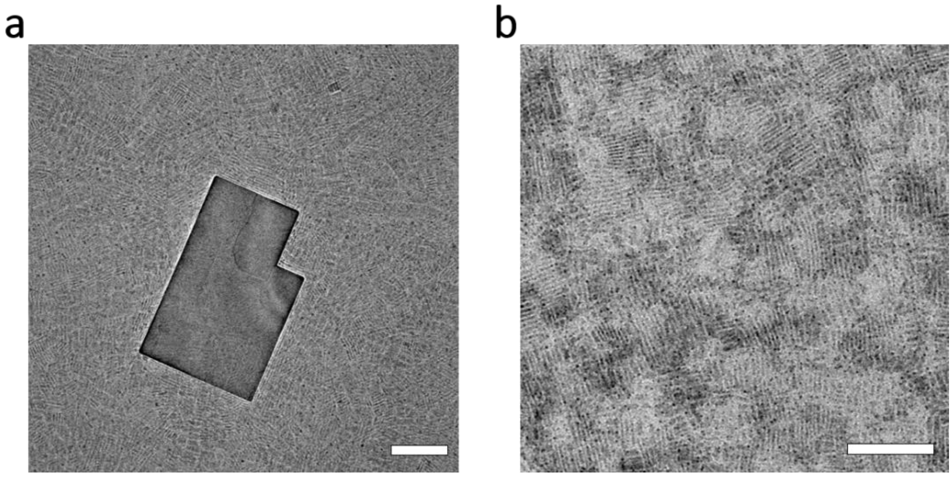

Figure S22. TEM images of the as-synthesized $CsPbBr_3$ NPL stacks after 1 month of storing the solution at (a) room temperature and (b) -4°C. Scale bars: 100 nm.

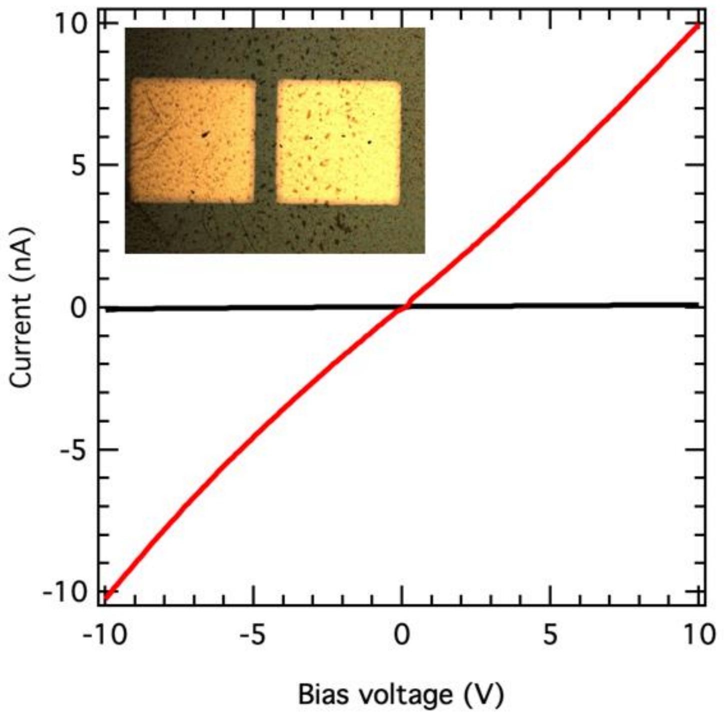

Figure S23. Electrical properties of heat-transformed NPL films. While pristine films prepared form fresh NPL solutions do not show stable conductance, the films transformed by heating at 110°C for 30 min manifest stable dark current (black), and a strong increase in current under illumination (red).

References

1. Simons, W. W. and Sadtler Research Laboratories (1978). *The Sadtler Handbook of Infrared Spectra*. Philadelphia, Pa: Sadtler Research Laboratories.
2. Lin-Vien, D.; Colthup, N. B.; Fateley, W. G.; Grasselli, J. G. (1991). Infrared and Raman Spectra of Commun Organic Compounds. In *The Handbook of Infrared and Raman Characteristic Frequencies of Organic Molecules* (pp. 423-454). San Diego: Academic Press.
3. Lin-Vien, D.; Colthup, N. B.; Fateley, W. G.; Grasselli, J. G. (1991). Compounds Containing $–NH_2$, –NHR, and $–NR_2$ Groups. In: *The Handbook of Infrared and Raman Characteristic Frequencies of Organic Molecules* (pp. 155-178). San Diego: Academic Press.
4. Dang, Z. Y.; Shamsi, J.; Palazon, F.; Imran, M.; Akkerman, Q. A.; Park, S.; Bertoni, G.; Prato, M.; Brescia, R.; Manna, L., In Situ Transmission Electron Microscopy Study of Electron Beam-Induced Transformations in Colloidal Cesium Lead Halide Perovskite Nanocrystals. *ACS Nano* **2017,** *11* (2), 2124-2132.
